# Ergonomics in laparoscopic surgery

**DOI:** 10.4103/0972-9941.65161

**Published:** 2010

**Authors:** Avinash N Supe, Gaurav V Kulkarni, Pradnya A Supe

**Affiliations:** Department of Surgical Gastroenterology, King Edward Memorial Hospital, Parel, Mumbai - 400 012, India; 1 Department of Gynaecology and Obstetrics, Sion Hospital, Mumbai - 400 023, India

**Keywords:** Ergonomics, human engineering, instrumentation environment

## Abstract

Laparoscopic surgery provides patients with less painful surgery but is more demanding for the surgeon. The increased technological complexity and sometimes poorly adapted equipment have led to increased complaints of surgeon fatigue and discomfort during laparoscopic surgery. Ergonomic integration and suitable laparoscopic operating room environment are essential to improve efficiency, safety, and comfort for the operating team. Understanding ergonomics can not only make life of surgeon comfortable in the operating room but also reduce physical strains on surgeon.

## INTRODUCTION

In 1901, when Kelling[1] introduced a visualising scope for the first time in the peritoneum of a dog, it was a landmark in the history of surgery. However, it took another eight decades for a perfected laparoscopic technique to be implemented, when for the first time, Mouret[2] performed a successful laparoscopic cholecystectomy in 1987.

In the wake of all advances, come the drawbacks. Laparoscopy is no exception. The drawbacks are majorly twofold. In the first scenario, the surgeon experiences the ill effects from the surgery, and secondly, the patient is the victim. This statement might seem superficial and poorly reflective in the first glance but it answers a much deeper question. There have been multiple reports of carpal tunnel syndrome, eyestrain and cervical spondylosis among unsuspecting surgeons performing multiple laparoscopic procedures in high-volume centres.[3] Reports of thenar neuropathy have arisen due to use of awkward thumb grips in case of laparoscopic pistol-grip instruments.[4]

In the first decade after the advent of laparoscopy, patients too have been found to be experiencing a lot of inconvenience with greater post-operative pain at port sites and due to other complications of the procedure in some cases. The mistakes leading to these poor outcomes seem to be completely avoidable with use of simple application of understanding of the physics and functioning of the whole event.

The operating room environment has been user-unfriendly traditionally and has recently been further complicated by the addition of complex machines and difficult interfaces between the patient and the surgeon.[5]

Herein we understand the principle of Ergonomics applicable to the field of laparoscopy as much as any other surgical expertise.

## WHAT IS ERGONOMICS?

The term ergonomics is derived from the Greek words "ergon" meaning work and "nomos" meaning natural laws or arrangement. Ergonomics is "the scientific study of people at work, in terms of equipment design, workplace layout, the working environment, safety, productivity, and training". Ergonomics is based on anatomy, physiology, psychology, and engineering, combined in a systems approach.

In simple words, it is the science of best suiting the worker to his job, or to make the setting and surroundings favourable for the laparoscopic surgeon. The term was formally defined in 1949 and has brought benefit and safety to many areas of human endeavor.[6]

The importance of ergonomics in the setting of laparoscopy cannot be over-emphasised. Studies have shown that correct ergonomics can reduce suturing time.[7] Pressure-related chronic pain among surgeons has been shown to be relieved by the use of ergonomically designed products.[8]

Hence, it is imperative to understand the applications of ergonomics for all surgeons practicing laparoscopy as well as novices yet to be initiated into this field. The aim of this article is to provide a basic understanding of the ergonomic challenges facing the laparoscopic surgeon and some simple modifications, which can go a long way in improving their operative practice.

## THE HAWTHORNE EFFECT

It has been a well-observed phenomenon that any individual performs a skill better and with more caution whenever he has the knowledge that he is under observation and assessment. This tends to skew the results towards more positive scores than would otherwise be obtained if the person under study was unaware of the assessment being performed. This constitutes the "Hawthorne effect" which has been found applicable to most scientific assessments of human function and hence an integral knowledge of this aspect is essential for ergonomic purposes.[9]

Laparoscopy, being a surgical skill performed by human dexterity and coordination, can definitely be assessed by ergonomic scales. Such assessments, though need to be done secretively to avoid the bias introduced by the Hawthorne effect, there would arise multiple ethical and analytical problems in doing so.

## ERGONOMIC CHALLENGES DURING LAPAROSCOPY

At the outset it is helpful to identify the potential problematic areas in the practice of minimal access surgery that pose unique ergonomic difficulties not faced by non-laparoscopic surgeons.

Differences between open and laparoscopic surgery are the following. Open surgery has a high degree of freedom and surgeons work in line with visual axis. There is a three-dimensional direct vision and direct tactile feedback. While during laparoscopic surgery there is a two-dimensional vision and loss of depth perception to some extent. There is fulcrum effect with tremor enhancement. There are only 4 degrees of freedom. The major limitation is that view is not under control of the surgeon.

The major factor unrelated to the skills which affect the efficiency of the surgeon is the decoupling of the visual and motor axes. There is also the loss of tactile feedback owing to substitution of instruments for the surgeon's hands. Visual orientation changes with the loss of depth perception due to indirect visual input and also the loss of peripheral vision caused by the limited viewing spectrum offered. The laparoscopic surgeon also assumes a relatively static posture during major part of the procedure which, ergonomically speaking, contributes to the inefficiency.

One of the most significant cognitive challenges for the general surgeon in his transformation into a laparoscopic surgeon is to overcome the spatial separation of the axis of vision and the axis of the physical aspect of the procedure. The surgeon does not get a chance to directly look at the instruments or his hands and also at the field of surgery at the same time. He has to learn to adapt to the difficulty of combining the two functions into the same-channelled approach in order to dexterously manipulate the tissues without direct contact. Studies have shown that working in separate coordinate systems decreases performance, leading to higher rates of error in the procedure.[10]

While learning the skills associated with open surgical procedures, as residents, we are trained to "see" with our hands as well as our eyes. We train our hands towards achieving this dual job in an attempt to reach the level of dexterity required to be competent. This constitutes the tactile feedback which is conspicuously lacking when one transitions from performing open procedures to laparoscopic surgeries. The long graspers manoeuvered through the trocars get substituted for the surgeon's hands and this definitely reduces the efficiency and increases the time of the dissection.[11]

Great concentration and skill is required for performing the complex laparoscopic surgeries. Hence, it has been observed that the operating surgeon assumes a more static posture during laparoscopic procedures compared to erstwhile open approach. These static postures have been demonstrated to be more disabling and harmful than dynamic postures are since muscles and tendons build up lactic acid and toxins when held for prolonged periods in same postures.[12-14]

Sensorial ergonomics (manipulations and visualisation) improve precision, dexterity, and confidence, while physical ergonomics provide comfort for surgeon. Together, these two elements of ergonomics increased safety, have better outcome and reduce the stress.[15]

## ERGONOMICS CONCEPTS IN OT

### OT position – American or European

A surgeon in two different positions can perform laparoscopic cholecystectomy. One is by standing on the left side of patients (preferred by Americans) and other is by standing between the legs (preferred by Europeans). Both the positions are convenient but some find one more ergonomically better. It is usually surgeon's preference or habit of getting adjusted to the position. Though port placement is similar, there is slight change in each position.

### OT table

Height of operating table should be adjusted between 64 and 77 cm above floor level since this discomfort and operative difficulty are lowest when instruments are positioned at elbow height.[16]

### Monitor position

Ergonomically, the best view for laparoscopy is with the monitor image at or within 25 optimal degrees below the horizontal plane of the eye.[17,18] This leads to least neck strain according to the available studies. Standard LCD monitors placed on a low cart separate from the operating room equipment may be used for best results. It is not advisable to have a "chin-up" arrangement on the part of the surgeon.[18] In operations where surgeons change their ports and positions, the second monitor is essential, e.g. total colectomy. Second monitor for assistants reduces strain on their neck.

### Trocar placements

**Figure 1:**
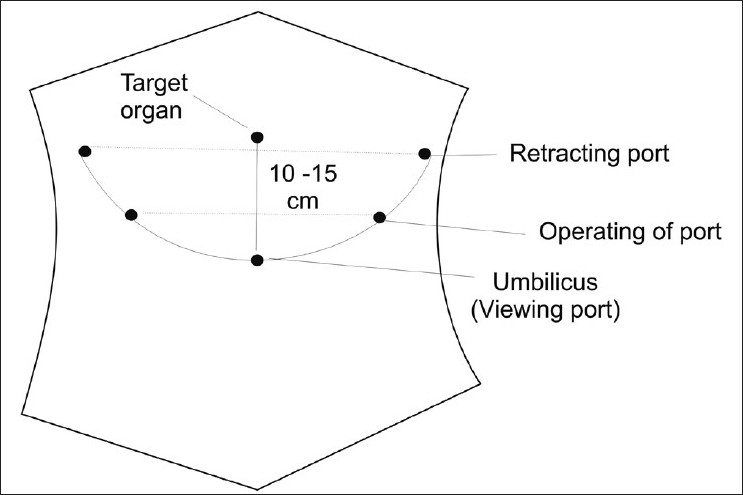
Port placement - Traingulation

There is no uniform consensus about port placements for advanced laparoscopic procedures. The placement of ports is currently dictated by the surgeons' preference based on individual experience. To facilitate smooth instrument manipulation along with adequate visualisation during laparoscopy, usually trocars are placed in triangular fashion. This is termed as triangulation [Figure 1]. The target organ should be 15–20 cm from the centre port used for placing the optical trocar. Generally, the two remaining trocars are placed in the same 15–20 cm arc at 5–7 cm on either side of the optical trocars. This allows the instruments to work at a 60°–90° angle[19] with the target tissue and avoids problems of long handle due to too far or too near placement of ports and the problem of abdominal wall interference. If necessary, two more retracting ports can be placed in the same arc but more laterally so that instruments do not clash.

**Figure 2:**
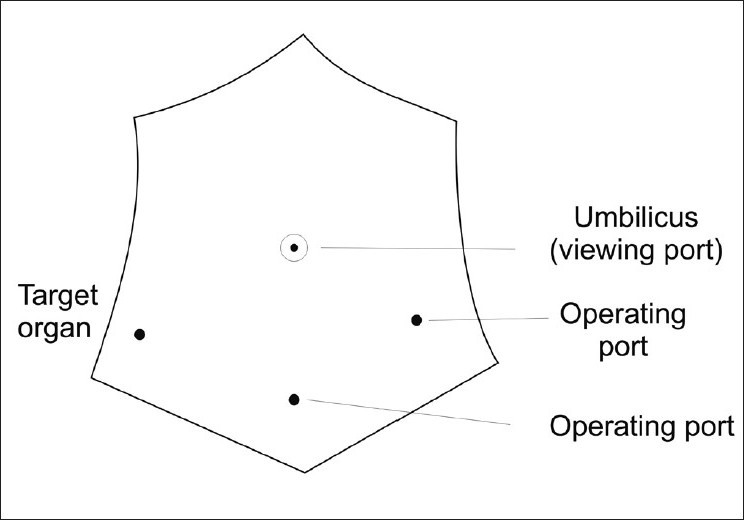
Port placement - Sectorisation

When optical trocar is placed as one of the lateral port trocar, it is called as sectorisation [Figure 2]. This is usually done during appendicectomy when 10 mm trocar is placed in subumbilical region as optical trocar. Two other trocars are placed below these trocars laterally. Sectoring of instruments should be avoided by beginners since it requires a greater degree of understanding and experience of the laparoscopic view and significantly different hand-eye coordination.

Another factor that one should consider during trocar placement is that the instrument length is limited. If trocar is too far from the desired position, then one has to push abdominal wall towards target organ to gain a few centimeters. This not only makes these movements less precise but also causes strain on the fingers and hand muscles. Similarly, if the angle between the target and instrument if too wide or obtuse, manipulation of curved instrument is very difficult. Most surgeons customise trocar position. If there is wrong placement of port, one has to push abdominal wall and your hands start paining.

Recently, GelPort laparoscopic systems have been introduced which restore spatial awareness and tactile feet while maintaining pneumoperitoneum.[20] It is possible to achieve rapid atraumatic manipulation, dissection, palpation and mobilisation of critical vessel by the surgeon's hand. This has been shown to lead to improved procedural times and also has given impetus to the concept of Hand Assisted Laparoscopic Surgery.

## MANIPULATION ANGLES FOR INSTRUMENTATION

Manasnayakorn *et al*.[21] have studied in animal models and have indicated that the best task efficiency and performance quality are obtained with an ideal manipulation angle between 45° and 60°. This can be achieved by correct placement of the ports. The 90 manipulation angle had the greatest muscle workload by the deltoid and trapezius of the extracorporeal and intracorporeal limbs and the extracorporeal dominant arm extensor and flexor groups. Manipulation angle ranging from 45° to 75° with equal azimuth angles is recommended. Manipulation angles below 45° or above 75° are accompanied by increased difficulty and degraded performance. Task efficiency was reported be better with equal azimuth angles than with unequal azimuth angles. Achieving equal azimuth angles may be difficult in many practical situations, but in principle, azimuth inequality should be avoided because it degrades task efficiency.

There exists a direct correlation between the manipulation and the elevation angles. With a manipulation angle of 60°, the corresponding optimal elevation angle which yields the shortest execution time and optimal quality performance is 60°. Wide manipulation angles necessitate wide elevation angles for optimal performance and task efficiency. When a 30° manipulation angle is imposed by the anatomy or build of the patient, the elevation angle should be also 30° as this combination carries the shortest execution time. The best ergonomic layout for endoscopic surgery consists of a manipulation angle ranging from 45° to 75° with equal azimuth angles.[22,23]

## EQUIPMENT RELATED CHALLENGES

The importance of Ergonomics in this field has been underscored by the US Food and Drug Administration reporting that probably half of the 1.3 million instrument-related injuries that occur in US hospitals each year could be due to poor instrument design.[24]

While performing minimal access surgery, the surgeon is typically viewing a two-dimensional video image of the operating field on a television video screen placed at a certain distance of 4–8 feet away from the surgeon's eye. Even with the best quality monitoring equipment, the quality and resolution detail of the image are not comparable with direct visualisation.

Another limiting aspect which the laparoscopist has to contend with is the loss of peripheral vision, which was one of the cornerstones of his surgical skills in open procedures. He is no longer permitted to view anything besides the immediate field of the operation and loses the luxury of efficient navigation in a larger surgical workspace.

The surgeon cannot move his instruments with unlimited degree of freedom. The laparoscopic instruments, which are most commonly in use in our setting at present, offer only 4 degrees of freedom of movement, which are rotation, up/down angulations, left/right angulations, in/out movement. Struges, Wright and Falk, *et al*. have suggested that an increase in degree of freedom from 4 to 6 increases dexterity by a factor of 1.5.[25]

Laparoscopic instruments work on reduced efficiency. For example, the laparoscopic grasper transmits force with a ratio of only 1:3 from the handle to the tip as compared to 3:1 with the hand-held hemostat. Hence, a laparoscopist has to work six times harder for similar results.[26] Moreover, these laparoscopic instruments are generally available in one standard size and hence surgeons of all heights, builds, and hand sizes work with same ones and the efficacy suffers somewhere along the way. Customised instruments are prohibitively costly.

Aggarwal *et al*.,[27] using the Imperial College Surgical Assessment Device (ICSAD) device, which generates objective scores of performance by analysing the movements of surgical instruments, proved that the newer generation 3D visual cameras significantly improved the laparoscopic precision of novices and experienced surgeons.

The skill-related factors which have a profound impact on the outcome are mostly related to the intracorporeal suturing techniques. These problems are a result of the necessity to suture in odd port positions in the absence of triangulation, suturing at odd angles to the tissue, suturing in the retroperitoneum and maintaining tension in continuous suturing while using less efficient instruments.

All the above problems associated with this skillful field of expertise are the specific challenges which laparoscopy presents.

### Mattern Waller handles

Mattern and Waller[28] have stated that improperly designed shapes of instruments cause strain on functional areas of the hand. They have designed handle that is based on ergonomic criteria. This multifunctional handle is shaped to fit only one hand and like a pistol handle, it rests continuously in the half-closed hand, similar to the ''basic position'' of the resting hand, between the ring and little fingers, with the thenar performing an encircling grip. The longitudinal axis of the instrument is an extension of the forearm's rotation axis. This allows pronation and supination to be transferred directly to the instrument effector.

### Single port laparoscopy

Single port laparoscopy has changed the concept of triangulation used in conventional laparoscopy. With single port, the instruments often cross each other, making the procedure "counter-counterintuitive". To overcome these difficulties, steerable endoscopes, bent and articulating instrumentation, magnetic anchorage and guidance systems as well as flexible robotics have been developed.

### Robotic surgery

Robotic surgery is ergonomically advantageous as it has 7 degrees of freedom as compared to laparoscopic hand surgery. This helps one to access deeper areas in abdomen such as oesophagus, pancreas and retroperitoneum. It also allows placements of ports in shorter arc without instrument interference.

## PHYSICAL CONSTRAINTS TO SURGEON DUE TO INEFFICIENT APPLICATION OF ERGONOMICS

Neck pain and spondylosis has been observed to be a recurring complaint among surgeons in high-volume centres in the first decade after the advent of minimal access surgery.[12] The same height, at which the video monitor used to be set for surgeons of different heights, was found to be the underlying cause.[12,13]

The other physical constraints reported are cervical spondylitis, shoulder pain due to abduction of shoulder (chicken wing scapula) during laparoscopy termed as "laparoscopic shoulder", backache, hand finger joint pain, tenosynovitis, burning eyes, stress exhaustion, and hand muscle injury.[23,29]

Most common reason for the inability of ergonomics to be applied optimally in the field of laparoscopy could be enumerated as the lack of complete awareness among surgeons, communication gap between the practitioners of laparoscopy and the designers of the instruments, inadequate knowledge of the potential problems for the users in the instruments created by the designers and the contradictory expert advice which reduces the credibility of ergonomics as a science.

The suggested position of arm is slightly abduction, retroversion and rotation inwards at shoulder level. The elbow should be bent at about 90°–120°. The surgeon has to remember that moving about and loosening up his hands intermittently is essential to prevent the buildup of lactic acid and ward off fatigue.[12] Problems related to depth perception, vision and loss of peripheral visual fields can be reduced by using a 10–15× magnification on the optical system offered by the recording camera and the output to the display. This can make life easier while operating, especially when dealing with minute and intricate internal anatomy.

Laparoscopic surgery provides patients with less painful surgery but is more demanding for the surgeon. The increased technological complexity and sometimes poorly adapted equipment have led to increased complaints of surgeon fatigue and discomfort during laparoscopic surgery. Better ergonomic integration and understanding ergonomics can not only make the life of surgeon comfortable in the operating room but also reduce physical strains on the surgeon.
